# *Arthrospira Platensis* (Spirulina) Supplementation on Laying Hens’ Performance: Eggs Physical, Chemical, and Sensorial Qualities

**DOI:** 10.3390/foods8090386

**Published:** 2019-09-02

**Authors:** Besma Omri, Marwen Amraoui, Arbi Tarek, Massimo Lucarini, Alessandra Durazzo, Nicola Cicero, Antonello Santini, Mounir Kamoun

**Affiliations:** 1 Laboratory of improvement and integrated development of animal productivity and food resources, Department of animal production, Higher School of Agriculture of Mateur, University of Carthage, Carthage Avenue de la République, P.O. Box 77, Amilcar, Tunis 1054, Tunisia; 2 CREA- Research Centre for Food and Nutrition, Via Ardeatina 546, 00178 Rome, Italy; 3 Dipartimento di Scienze biomediche, odontoiatriche e delle immagini morfologiche e funzionali, Università degli Studi di Messina, Polo Universitario Annunziata, 98168 Messina, Italy; 4 Department of Pharmacy, University of Napoli Federico II, Via D. Montesano, 49-80131 Napoli, Italy

**Keywords:** cholesterol, egg quality, Haugh unit, spirulina, yolk color

## Abstract

The present study evaluated the effects of dietary supplementation of spirulina on laying hens’ performances: Eggs’ physical, chemical, and sensorial qualities. A total of 45 Lohman White hens, 44 weeks of age, were randomized into 3 groups of 15 birds. Hens were given 120 g/d of a basal diet containing 0% (control), 1.5%, and 2.5% of spirulina for 6 weeks. Albumen height and consequently Haugh unit were significantly affected by dietary supplementation of spirulina (*p* < 0.05) and by weeks on diet (*p* < 0.05). This supplement did not affect (*p* > 0.05) egg yolk weight or height. However, spirulina increased egg yolk redness (a*) from 1.33 (C) to 12.67 (D1) and 16.19 (D2) and reduced (*p* < 0.05) the yellowness (b*) parameter from 62.1(C) to 58.17 (D1) and 55.87 (D2). Egg yolks from hens fed spirulina were darker, more red, and less yellow in color than egg yolks from hens fed the control-diet (*p* < 0.0001). However, spirulina did not affect (*p* > 0.05) egg yolks’ total cholesterol concentration. In conclusion, a significant enhancement of egg yolk color was found in response to spirulina supplementation. Further investigations are needed to evaluate the impact of spirulina on egg yolks’ fatty acids profile.

## 1. Introduction

*Arthrospira platensis* (spirulina) is a filamentous spiral-shaped blue-green algae [1,2]. It has been recognized as a genus of photosynthetic bacteria (*Arthrospira*). This microorganism belongs to the class of Cyanophyta/Cyanobacteria that grow naturally in warm and alkaline aquatic media. From the perspective of a nutraceutical view [3,4,5,6,7,8], spirulina is considered as a functional food due to its high protein content (65% to 70% dry matter), high amount of vitamin and mineral content, and wide variety of natural carotene and xanthophyll phytopigments [9,10], and it is generally regarded as safe (GRAS) by the European Food Safety Authority (EFSA) [11]. Spirulina is a source of other nutritionally beneficial organic molecules, such as gamma linoleic acid, phenolic acids, and chlorophyll [12,13]. Deng et al. [14] and Bashandy et al. [15] reported that spirulina has many health benefits, including antioxidant properties, hypolipidemic action, and immunostimulating or anti- inflammatory effects [16,17]. These properties have been verified using laboratory animals [18,19]. Spirulina was used as alternative dietary sources in poultry diets [20]. In these diets, spirulina can be used up to 10% as a partial replacement of conventional proteins without any adverse effects [21]. Dietary vitamin and mineral premixes can be omitted when spirulina algae are included in chicken rations [22], due to its nutrient-rich composition. Zeweil et al. [23] reported that dietary supplementation of spirulina in chickens under heat stress conditions could decrease adverse effects of chronic heat stress on growth performance and immunity of a Gimmizah local strain of chicken. Spirulina could also be used as an effective way to improve the poultry product quality to meet consumer preferences [24], owing to its high concentration of carotenoids [25]. Zahroojian et al. [26] and Mariey et al. [12] found that dietary inclusion of spirulina at a concentration of 2% to 2.5% in laying hens’ feed intensified egg yolk color to make it more aesthetically pleasing for consumers. This coloration intensification is thought to be due to spirulina’s high concentration of β-carotene [27]. Dietary incorporation of spirulina can also reduce egg yolk total cholesterol and saturated fatty acid content and increase its omega-3 polyunsaturated fatty acids levels [28,29]. According to Zahroojian et al. [26], it has been shown that addition of spirulina at a level of 2% to 2.5% in the laying hen diet was associated with a significant increase of egg yolk color determined by comparison with the BASF Ovo-Color Fan, an Ovo-Color Yolk Fan supplied by BASF (Florham Park, NJ, USA). However, egg yolk color estimation using the color measuring device, Chroma Meter, was not reported. Therefore, the objective of the present study was to evaluate the effect of dietary incorporation of spirulina on laying hens’ performances, egg physical characteristics, egg yolk color, and total cholesterol concentration.

## 2. Materials and Methods 

### 2.1. Diet Preparation

Two kilos of spirulina (*Spirulina platensis*) were purchased from a regional producer located in the region of Gabes (Tunisia). A standard diet (control diet) (C) for laying hens based on corn and soybean meal and 2 supplemented diets, designated as follows: 1.5% of spirulina-supplemented diet (D1) and 2.5% of spirulina-supplemented diet (D2) were individually prepared by mixing the control diet (C) thoroughly with the designated supplements at the required incorporation levels as shown in Table 1. 

### 2.2. Ethical Considerations

All procedures related to animals’ care, handling, and sampling were conducted under the approval of the Official Animal Care and Use Committee of the Higher School of Agriculture of Mateur (protocol N°05/15) before the initiation of the study and followed the Tunisian guidelines.

### 2.3. Experimental Design

Forty-five 44-week-old *Lohman White* laying hens were divided randomly into 3 groups of 15 birds. Each group was allocated to one of the three following dietary treatments: (1) Control diet (C), (2) 1.5% of spirulina-supplemented diet (D1), and (3) 2.5% of spirulina-supplemented diet (D2). Each hen was fed daily 120 g of a basal diet containing 0% (control), basal diet plus 1.5% g of spirulina, or basal diet plus 2.5% g of spirulina. The ingredients and chemical composition of the diets are shown in Table 1. Hens were housed in cages with individual feed troughs and common water troughs in a room with ambient temperature of about 20 °C and a photoperiod of 16 h light:8 h darkness cycle. Water was provided ad libitum throughout the trial period, which lasted 42 days.

### 2.4. Data Collection

All the birds were weighed individually at the beginning and the end of the experiment to determine the live weight changes. Feed was offered once daily at 7:30 a.m. and refusal was measured weekly. Egg production and weight were recorded daily. Daily feed consumption, laying rate (number of laid eggs × 100/number of feeding days), and feed conversion ratio (feed consumption/number of eggs × egg weight) were calculated per week. 

Eggs laid during the 1st, 14th, 21st, 28th, 35th, and 42nd days were used for egg physical characteristics measurements (egg albumen, yolk and shell weights, albumen and yolk heights, Haugh unit, yolk diameter, yolk index, and shell thickness) and egg yolk color using the color measuring device Konica Minolta Chroma Meter CR- 400/410 (Minolta Corp.) according to the CIE (Commission Internationale de L’Eclairage) L * (lightness: negative towards black, positive towards white), a * (redness: negative towards green, positive towards red), and b * (yellowness: negative towards blue, positive towards yellow) color system and colorimetric interval, dL * (Lightness interval), da * (Red/Green interval), and db * (Yellow/Blue interval), between the spirulina-supplemented diet (D1 and D2) and control diet (C). The instrument was set perpendicular to the egg yolk surface in a Petri dish. The parameters, L *, a *, and b *, were measured three times and the final values were calculated as the averages of the three corresponding values measured.

Haugh unit (UH) was calculated according to the formula [31]:

Haugh unit = 100 × log (HA − 1.7 × W0.37 + 7.6),

where: HA = albumen height (mm) and W = egg weight (g).

Yolk index was calculated according to the formula:

Yolk index = Yolk height (mm)/Yolk diameter (mm).

Shell thickness, albumen and yolk heights, and yolk diameter were measured using a caliper.

Eggs laid during days 1 and 28 of the experimental period were pooled per hen and used for egg yolk total cholesterol determination.

### 2.5. Chemical Analysis

The dry matter of diets (DM) was determined at 105 °C for 24 h. All other analyses were done on samples dried at 65 °C and ground in a mill to pass through a 0.5-mm screen. Ash content was determined by igniting the ground sample at 550 °C in a muffle furnace for 10 h. The Association of Official Analytical Chemists method [32] was used for crude protein (CP) determination. 

Egg yolk samples pooled per hen were solubilized in 2% (w/v) NaCl solution [33] and used for cholesterol determination using standard enzymatic-colorimetric methods (cholesterol enzymatic colorimetric test, CHOD-PAP Biomaghreb, Tunisia).

### 2.6. Statistical Analysis

Data of repeated measurements (feed refusal and intake, laying rate, egg mass, feed conversion ratio, egg physical characteristics, yolk total cholesterol, and yolk color traits) were tested for diet, week on diet effects, and their interaction using mixed models with compound symmetry covariance structures of SAS (Statistical Analysis System) [34]. 

Data of the hens’ live weight change were tested for diet effect using the general linear model (GLM) procedure of the Statistical Analysis System (SAS) [34] according to the following model:

Yij=u+Ai+eij,

where:

Yij= represents the *j*th observation on the *i*th treatment;
μ = overall mean;
Ai= main effect of the *i*th treatment;
eij= random error present in the *j*th observation on the *i*th treatment.

## 3. Results

### 3.1. Laying Performance

Laying hens’ performances are shown in Table 2. All hens showed a loss of body weight at the end of the experimental period, but this weight loss was not affected (*p* > 0.05) by dietary treatment. Feed refusal and consumption was not affected (*p* > 0.05) by dietary addition of spirulina and did not change (*p* > 0.05) over the weeks on the diet. In parallel, the laying rate and consequently daily egg mass production were not affected (*p* > 0.05) by dietary treatment and weeks on the diet and their interaction. Only egg weight was affected (*p* < 0.05) by dietary treatment. Supplementation of 2.5% spirulina (D2) increased (*p* < 0.05) egg weight from 62.76 ± 1.53 g to 64.33 ± 1.83 g. The feed conversion ratio (FCR) was not affected (*p* > 0.05) by dietary treatment, weeks on diet, and their interaction.

### 3.2. Egg Physical Characteristics

The determined physical characteristics of eggs (egg, yolk, albumen and shell weights, yolk and albumen heights, yolk diameter, UH (Haugh Unit), and yolk index) are shown in Table 3. 

Ours results showed that egg and albumen weights were affected (*p* < 0.05) by dietary inclusion of spirulina. Mean egg weight varied from 62.22 ± 2.98 g (C) to 64.43 ± 3.04 g (D2). Albumen weight of hens fed on 2.5% spirulina was the highest, with mean values of 36.20 ± 2.1 g vs. 35.08 ± 2.5 g (D1) and 34.41 ± 1.81 g (C). Shell thickness was affected by dietary treatment (*p* < 0.05), weeks on diet (*p* < 0.0001), and their interaction (*p* < 0.0001).

Albumen height and consequently Haugh unit were significantly affected by dietary supplementation of spirulina (*p* < 0.05) and by weeks on diet (*p* < 0.05). Dietary incorporation of spirulina did not affect (*p* > 0.05) egg yolk weight and height. However, these parameters were influenced (*p* < 0.05) by weeks on diet. Egg yolk diameter and index were affected by weeks on diet (*p* < 0.0001) and the interaction, treatment*week on diet. 

Concerning albumen height, UH, and shell thickness, for each diet, differences between parameters at week 1 and their average mean at week 3 and week 6, as well as differences between means at week 3 and week 6, were compared (Table 4). Tested differences of albumen height were significant (*p* < 0.05) for the control diet, indicating an increase of albumen height at week 1. For each of the D1- and D2-diets, only differences between heights at week 3 and week 6 were significant (*p* < 0.05), indicating an increase of albumen height at week 6. UH did not change (*p* < 0.05) over time for the control, decreased (*p* > 0.05) at week 3 for the D1-diet, and increased (*p* > 0.05) for the D1-diet and D2-diet at week 6. Shell thickness increased (*p* > 0.05) for the D2-diet at week 1, week 3, and week 2 and for the D2-diet at week 6. 

### 3.3. Egg Yolk Color

Egg yolk color traits determined by a Konica Minolta Chroma Meter CR-410 were affected by dietary treatment (*p* < 0.0001), weeks on diet (*p* < 0.0001), and their interaction (*p* < 0.0001) (Table 5). Hens fed the control diet had the highest (*p* < 0.0001) lightness L*, with a mean value of 70.55 vs. 65.98 and 63.74 corresponding to hens fed with 1.5% and 2.5% spirulina, respectively. These values showed that the egg yolk of the control group was characterized by an intense yellow color. 

Dietary supplementation of spirulina increased egg yolk redness, a*, from 1.33 ± 2.34 (C) to 12.67 ± 8.94 (D1) and 16.19 ± 9.85 (D2). The redness mean value (a*) corresponding to the control group indicated that this group had a weak red hue. Concerning the yellowness (b*), hens fed the diet without spirulina supplementation had the highest mean value (62.1 ± 2.66), which indicated that egg yolk color was sufficiently intense. Dietary inclusion of spirulina resulted in a significant decrease (*p* < 0.0001) of the egg yolk yellowness (b*). In fact, hens fed 2.5% and 1.5% spirulina had a yellowness mean value of 55.87 ± 3.93 and 58.17 ± 3.41, respectively.

The colorimetric interval between the spirulina-supplemented diet and control diet are represented in Table 6. These colorimetric intervals, dL*(lightness interval), da*(red/green interval), and db*(yellow/blue interval), between the spirulina-supplemented diet and control diet (C), determined by a Chroma Meter, showed that egg yolks from hens fed spirulina were darker, more red and, less yellow in color than egg yolks from hens fed the C-diet (*p* < 0.0001). It was found that egg yolk color parameters (L*, a *, and b*) changed over time for all diets (Table 7). L* decreased (*p* < 0.0001) in the C-diet, D1-diet, and D2-diet after the first week and increased (*p* < 0.0001) at week 3 and 6 for the D1-diet and D2-diet. However, egg yolk lightness decreased (*p* < 0.001) at the sixth week for the control diet. Concerning egg yolk redness (a*), a significant increase (*p* < 0.0001) was found at week 1 for the three diets. This increase was recorded during the experimental period for the D1 and D2 differences between the mean a* values at week 3 and were significant (*p* < 0.0001), indicating a decrease of egg yolk redness. Egg yolk yellowness increased (*p* < 0.0001) at week 1, week 3, and week 6 for C-, D1-, and D2-diets. By contrast, differences between the mean b* values for D2 at week 3 and for C at week 6 were negative (*p* < 0.0001), indicating a decrease of egg yolk yellowness.

### 3.4. Egg Yolk Cholesterol Concentration

The effect of dietary supplementation of 1.5% and 2.5% spirulina on egg yolk total cholesterol concentration is represented in Table 8.

Our data showed that egg yolk concentration of total cholesterol was only affected (*p* < 0.05) by the weeks on diet. However, egg yolk concentration of total cholesterol was 14.35 ± 0.88 mg/g for hens fed the C-diet vs. 13.89 ± 1.21 and 14.39 ± 1.23 mg/g for hens fed 1.5% and 2.5% spirulina, respectively.

## 4. Discussion

### 4.1. Laying Performances

Spirulina inclusion was without impact on feed consumption. Our results are in agreement with those reported by Dogan et al. [35], who reported that dietary addition of 0.5%, 1%, and 2% of spirulina did not affect feed consumption of laying quails.

In the present study, hens’ live body weight losses were 56.00 vs. 19.33 g/42 d and laying rates were high (92.19% vs. 96.38%) and not affected by spirulina inclusion. Hens were fed ad libitum before our experimental study and then feed was restricted to 120 g/d so that animals showed this loss of body weight throughout the experimental period. Furthermore, only egg weight was significantly increased from 62.76 (C) to 63.18 g (1.5% spirulina) and 64.33g (2.5% spirulina). This increase of egg weight could be attributed to the high protein content in spirulina. These results are partially in agreement with those reported by Mariey et al. [12], who found that hens’ live weight of Sinai (S) and Gimmizah (G) was not affected by dietary supplementation of 0.1%, 0.15%, and 0.2% spirulina. However, this supplementation decreased the feed conversion ratio. The lowest value was attributed to hens fed 0.2% spirulina; 3.46 versus 4.54 for the control group. Dogan et al. [35] also reported that dietary inclusion of 0.5%, 1%, and 2% spirulina did not affect the laying rate, feed conversion ratio, and egg weight. By contrast, Selim et al. [36] found that inclusion of 0%, 0.1%, 0.2%, and 0.3% spirulina increased hens’ final weight from 1222 g (0%) to 1227 (0.1%), 1238 (0.2%), and 1253 g (0.3%). However, Mariey et al. [12] reported that dietary incorporation of 0.1%, 0.15%, and 0.2% spirulina increased the laying rate, egg weight, and egg mass. Zahroojian et al. [26] showed that addition of 1.5%, 2%, and 2.5% spirulina in the diet of 128 Hy-line White hens did not affect the laying rate and egg weight. This absence of changes in laying hens’ performances associated with the use of spirulina might be attributed to the rate of inclusion of this alga, the variety, cultural practices, and climate.

### 4.2. Egg Physical Characteristics

With the exception of egg and albumen weights, albumen height, UH, and shell thickness, egg characteristics were not affected (*p* > 0.05) by dietary treatment and observed changes over time were numerically small and, therefore, of little physiological significance. Selim et al. [36] reported that dietary addition of 0.1%, 0.2%, and 0.3% spirulina did not affect egg physical characteristics (albumen index, albumen, yolk and shell weights, yolk index, and Haugh unit) determined at the end of the fourth week of the experiment trial. However, hens fed 0.1%, 0.2%, and 0.3% spirulina had a thicker shell of 0.356, 0.401, and 0.423 mm compared to those fed the control diet (0.314 mm). This finding could be attributed to the high calcium content of spirulina. Concerning the Haugh unit, Parisse [37] reported that eggs with a Haugh unit higher than 70 are considered excellent eggs, eggs with 70 to 60 Haugh units are acceptable, while eggs with Haugh units below 60 are of poor quality. Our results showed that hens fed 1.5% spirulina had the highest Haugh unit, with a mean value of 97.28 versus 95.99 (2.5%) and 93.33 (0%). Mean values of the present study were higher than 70, thus our eggs may be considered as excellent eggs. This high Haugh unit could be attributed to the fact that this parameter was determined on each egg laid on the same day and not on stored eggs. The pigment content of the supplemented spirulina could be responsible for the observed difference between treatments.

Mariey et al. [12] reported that dietary supplementation of spirulina at 0.1%, 0.15%, and 0.2% did not affect shell weight, albumen percentage and index, and Haugh's unit. Our data showed that dietary inclusion of spirulina did not affect egg yolk weight, height, diameter, and index. These results were not in agreement with those reported by Dogan et al. [35], who found that incorporation of 2% spirulina increased the egg yolk index of laying quails from 47.48 to 48.45 mm, shell weight from 1.55 to 1.68 g, and shell thickness from 0.199 to 0.207 mm. Mariey et al. [12] also reported that dietary supplementation of 0.15% spirulina increased egg yolk weight from 31.10 to 32.90 g. 

By contrast, Zahroojian et al. [26] reported that incorporation of 1.5%, 2%, and 2.5% spirulina did not affect eggs’ physical qualities (yolk index, Haugh unit, shell thickness, shell weight, and specific gravity)

### 4.3. Egg Yolk Color

Egg choice by consumers is no longer only based on yolk cholesterol content or fatty acids profile but also on its color [38]. The required degree of pigmentation varies among and within countries, but golden to yellow colors are usually considered more attractive [39]. Egg yolk color intensification can be achieved by dietary supplementation of carotenoids. However, laying hens are unable to synthesize these pigments. They need to be provided in their diet’s ingredients [40]. Carotenoids are synthesized by algae, plants, fungi, and some bacteria. In the present study, egg yolk color intensification was achieved with dietary incorporation of spirulina. Concerning egg yolk color evaluation, it was estimated by colorimetric determination of lightness (L *), redness (a *), and yellowness (b*) indexes and colorimetric intervals (ΔL *, Δa *, Δb *).

Our results showed dietary supplementation of spirulina increased egg yolk redness and reduced yolk yellowness. Studies on the effect of spirulina on egg yolk color traits measured by a Chroma Meter are lacking. However, Mariey et al. [12] reported that dietary incorporation of 0.1%, 0.15%, and 0.2% spirulina increased the egg yolk color score (RYCF) from 6.3 (0.1%) and 6.7 (0.15%) to 7.6 (0.2%). Zahroojian et al. [26] also found that supplementation of 1.5%, 2%, and 2.5% spirulina increased the egg yolk color score from 10.55 (1.5%) and 11.43 (2%) to 11.66 (2.5%) compared to the control. Dietary inclusion of 0.1%, 0.2%, and 0.3% spirulina increased the egg yolk color score from 6.11 (0.1%) to 6.89 (0.2%) and 7.33 (0.3%) [36]. Anderson et al. [27] also evaluated the effect of dietary addition of 0.25%, 0.5%, 1.2%, and 4% spirulina, which increased quail egg yolk color measured at the 2nd and 23rd day of treatment. Park et al. [41] reported that incorporation of marine microalgae (*schizochytrium*) at 0.5% and 1% in laying hens’ diet increased the egg yolk color score after 6 weeks of treatment, with a mean value of 9 and 8.8 compared to 8.7 corresponding to the control group. 

### 4.4. Egg Yolk Cholesterol Concentration 

Dietary supplementation of spirulina did not affect the egg yolks’ total cholesterol concentration. The absence of the effect of spirulina on egg yolk total cholesterol was in agreement with the results reported by Zahroojian et al. [26], who reported that dietary addition of 1.5%, 2%, and 2.5% spirulina did not affect the egg yolk concentration of cholesterol, with mean values of 10 (1.5%), 10.59 (2%), and 11.81 mg/g (2.5%). By contrast, Dogan et al. [35] reported a reduction in egg yolk cholesterol concentration per gram of yolk from 19.65 to 18.93 when laying hens’ diet were supplemented with 1% and 2% spirulina. Mariey et al. [12] also reported that egg yolk concentration of cholesterol decreased from 13.50 to 10.20 mg/g with dietary addition of 0.2% spirulina. Total egg cholesterol also decreased from 12.9 to 9.9 mg/g when spirulina was supplemented at a level of 0.3% [28]. 

Selim et al. [36] reported that dietary incorporation of 0.1%, 0.2%, and 0.3% spirulina reduced egg yolk cholesterol concentration from 13.6 mg/g (control) to 13.1 (0.1%), 12.4 (0.2%), and 11.7 mg/g (0.3%). Park et al. [41] also found that incorporation of *schizochytrium*, a marine microalgae, in laying hens’ diets at a level of 0.5% and 1% reduced serum cholesterol from 133.8 to 118.5 mg/dl. These authors attributed this reduction to the high contents of polyunsaturated fatty acids in spirulina. In fact, omega-3 fatty acids stimulate the activity of LCAT (lecithin cholesterol acyltransferase) [42], an enzyme responsible for the serum cholesterol esterification [43], so that most newly formed cholesterol esters are initially incorporated in HDL (High-Density Lipoprotein) [44]. 

Chen et al. [45] also reported that the docosahexanoic acid (DHA) of microalgae may inhibit the activity of 3-hydroxy-3-methylglutaryl-coenzyme A (HMG-CoA) reductase by reducing cholesterol synthesis so that the serum cholesterol concentration decreases. 

It can be concluded that incorporation of spirulina in 44-week-old Lohman White laying hens’ diets was without effects on laying hens’ performances and increased egg weight, shell thickness, albumen weight and height, and Haugh unit. The use of *Spirulina platensis* as a laying hens’ feed additive increased egg yolk color, as measured by a chromameter, and did not affect the total cholesterol concentration. Further investigations are needed to evaluate the impact of spirulina on egg yolk 

## Figures and Tables

**Table 1:** Ingredients and chemical composition of diets.

	Treatment		
Ingredients, %	C	D1 (1.5% spirulina)	D2 (2.5% spirulina)
Yellow corn	66.5	65.5	65.5
Soybean meal	25.5	25	24
Calcium carbonate, Mineral and Vitamin mixtureα	8	8	8
spirulina	0	1.5	2.5
Chemical Composition			
Dry Matter (%)	90.63	89.73	90.73
Organic Matter (% DM)	80.54	79.49	80.73
Crude Protein (% DM)	16.0	16.19	17.3
Ether Extract (% DM)	3.2	3.4	3.2
NDF (% DM)	10.15	10.5	11
¥ Metabolizable Energy, kcal/kg DM	2732.32	2738.42	2718.67

αControl (C) provided following nutrients per 100 g: Ca, 4.3 g; P, 0.6 g; Na, 0.14 g; Cl, 0.23 g; Fe, 4 mg; Zn, 40 mg; Mn, 7 mg; Cu, 0.3 mg; I, 0.08 mg; Se, 0.01 mg; Co, 0.02; methionine, 0.39 g; methionine + cysteine, 0.69 g; lysine, 0.89 g; Retinol, 800 IU; Cholecalciferol, 220 IU; α-tocopherol, 1.1 IU; Thiamin, 0.33 IU; Nicotinic acid, 909 IU. ¥ Metabolizable Energy = 2707.71 + 58.63 * EE−16.06 * NDF [30].

**Table 2:** Effect of spirulina on hens’ live weight changes and laying performances.

	Treatment			SEM ^&^	*p*-value		
	C ^α^	D1 ^α^	D2 ^α^		Trt ^β^	W *^β^*	Trt * W *^β^*
LW change, (g/42 days)	−56.00 ± 101.05	−19.33 ± 120.9	−30.00 ± 139.01	31.30	NS	−	−
Feed intake, g DM/hen/day	105.11 ± 3.89	106.79 ± 0.71	105.43 ± 4.09	0.66	NS	NS	NS
Feed Refusal, g DM/hen/day	3.65 ± 3.89	2.88 ± 0.71	3.44 ± 4.09	0.66	NS	NS	NS
Laying rate, %	96.38 ± 4.18	94.67 ± 4.19	92.19 ± 9.41	1.28	NS	NS	NS
Egg weight, g	62.76 b ± 1.53	63.18b ± 1.47	64.33a ± 1.83	0.32	*	NS	NS
Egg mass, g/hen/day	60.47 ± 2.57	59.80 ± 2.83	59.34 ± 6.58	0.88	NS	NS	NS
Feed conversion ratio	1.74 ± 0.089	1.78 ± 0.082	1.79 ± 0.23	0.03	NS	NS	NS

^α^**C** = control diet with 0% of spirulina; ^α^ D1 = control diet supplemented with 1.5% of spirulina; ^α^ D2 = control diet supplemented with 2.5% of spirulina; and SEM = standard error of the mean; *^β^* trt = treatment; *^β^* W = week; *^β^* trt * W = treatment – week interaction; * = *p* < 0.05; NS = *p* ≥ 0.05; ^ab^: Mean in the same row with different superscripts are significantly different (*p* < 0.05).

**Table 3:** Effect of spirulina on egg physical characteristics.

	Treatment			SEM ^&^	*p*-value		
	C ^α^	D1 ^α^	D2 ^α^		Trt ^β^	W ^β^	Trt * W ^β^
Egg weight, g	62.22 ^b^ ± 2.98	62.98^ab^ ± 3.54	64.43 ^a^ ± 3.04	0.54	*	NS	NS
Yolk weight, g	17.92 ^a^ ± 1.41	18.1 ^a^ ± 1.17	18.22 ^a^ ± 1.23	0.22	NS	*	NS
Albumen weight, g	34.41 ^b^ ± 1.81	35.08 ^ab^ ± 2.5	36.20 ^a^ ± 2.71	0.4	*	NS	NS
Shell weight, g	6.63 ^a^ ± 0.41	6.55 ^a^ ± 0.41	6.75 ^a^ ± 0.46	0.07	NS	*	*
Shell thickness, mm	0.39 ^ab^ ± 0.05	0.41 ^a^ ± 0.06	0.38 ^b^ ± 0.04	0.008	*	***	***
Yolk height, mm	18.10 ^a^ ± 0.97	18.59 ^a^ ± 1.14	18.50 ^a^ ± 1.08	0.18	NS	***	*NS*
Albumen height, mm	8.64 ^b^ ± 0.79	9.51^a^ ± 0.94	9.24 ^a^ ± 0.93	0.15	*	***	*NS*
Yolk diameter, mm	42.15 ^a^ ± 1.55	42.55 ^a^ ± 1.52	42.39 ^a^ ± 1.68	0.27	NS	***	***
UH	93.22 ^b^ ± 3.92	97.28 ^a^ ± 4.39	95.99 ^a^ ± 4.32	0.71	**	*	*NS*
Yolk index	0.43 ^a^ ± 0.025	0.43 ^a^ ± 0.027	0.44 ^a^ ± 0.031	0.005	NS	***	*

^α^**C** = control diet with 0% of spirulina; ^α^ **D1** = control diet supplemented with 1.5% spirulina; ^α^ **D2** = control diet supplemented with 2.5% spirulina; **SEM** ^&^ = standard error of the mean; *^β^* trt = treatment; *^β^* W = week; *^β^* trt * W = treatment–week interaction; *** = *p* < 0.0001; ** = *p* < 0.001; * = *p* < 0.05; NS = *p* **≥** 0.05; ^ab^: Mean in the same row with different superscripts are significantly different (*p* < 0.05).

**Table 4:** Week effect of the diet’s distribution on albumen height, UH (Haugh Unit), and shell thickness.

	Diets		Mean of Difference		
		Actual Mean at Week 1	Week 1 and Subsequent Weeks	Week 3 and Subsequent Weeks	Week 3 and Week 6
Albumen height, mm	^α^ C	8.02	1.49 *	0.56 ^NS^	−0.09 ^NS^
	^α^ D1	8.67	0.66 ^NS^	−0.25 ^NS^	0.44 **
	^α^ D2	8.83	0.45 ^NS^	0.5 ^NS^	0.73 ***
UH	^α^ C	90.90	−2.1 ^NS^	1.6 ^NS^	6.7NS
	^α^ D1	93.19	−3.76 ^NS^	−1.65 *	5.17 *
	^α^ D2	93.91	−1.03 ^NS^	0.7 *	6.1 ***
Shell thickness, mm	^α^ C	0.46	0.03 ^NS^	−0.016 ^NS^	−0.006 ^NS^
	^α^ D1	0.42	0.008 ^NS^	0.016 ^NS^	0.11 *
	^α^ D2	0.42	0.01 *	0.1 **	0.3 ***

^α^**C** = control diet with 0% spirulina; ^α^ **D1** = control diet supplemented with 1.5% spirulina; ^α^ **D2** = control diet supplemented with 2.5% spirulina; *** = *p* < 0.0001; ** = *p* < 0.001; * = *p* < 0.05; NS = *p* ≥ 0.05.

**Table 5:** Effect of spirulina on egg yolk color.

	Treatment			SEM ^&^	*p*-value		
	C ^α^	D1 ^α^	D2 ^α^		Trt *^β^*	W *^β^*	Trt *W *^β^*
L *	70.55^a^ ± 1.17	65.98^b^ ± 4.42	63.74^c^ ± 5.07	0.67	***	***	***
a *	1.33^b^ ± 2.34	12.76^a^ ± 8.94	16.19^a^ ± 9.85	1.32	***	***	***
b *	62.10^a^ ± 2.66	58.17^b^ ± 3.41	55.87^c^ ± 3.93	0.57	***	***	***

**^α^** C = control diet with 0% spirulina; **^α^** D1 = control diet supplemented with 1.5% spirulina; **^α^** D2 = control diet supplemented with 2.5% spirulina; SEM ^&^ = standard error of the mean; *^β^* trt = treatment; ^β^ W = week; ^β^ trt * W = treatment–week interaction; *** = *p* < 0.0001; ^a,b,c^: Means in the same row with different superscripts are significantly different (*p* < 0.05); L*: lightness, a*: redness and b*: yellowness.

**Table 6:** Colorimetric interval between the spirulina-supplemented diet and control diet.

	Treatment		SEM ^&^	*p*-Value		
	C^α^ and D1 ^α^	C ^α^ and D2 ^α^		Trt *^β^*	W *^β^*	Trt *W *^β^*
ΔL*	−4.57 ^a^ ± 4.25	−6.81 ^b^ ± 5.07	0.65	***	***	***
Δa*	11.43 ^b^ ± 8.22	14.86 ^a^ ± 9.85	1.37	***	***	***
Δb*	−3.93 ^a^ ± 3.41	−6.23 ^b^ ± 3.94	0.57	***	***	*

**^α^** C = control diet with 0% spirulina; **^α^** D1 = control diet supplemented with 1.5% spirulina; **^α^** D2 = control diet supplemented with 2.5% spirulina; SEM **^&^** = standard error of the mean; *^β^* trt = treatment; *^β^* W = week; *^β^* trt*W = treatment–week interaction; *** = *p* < 0.0001; * = *p* < 0.05; ^a,b^: Means in the same row with different superscripts are significantly different (*p* < 0.05).

**Table 7:** Week effect of the diet´s distribution on egg yolk color.

	Diets		Mean of Difference		
		Actual Mean at Week 1	Week 1 and Subsequent Weeks	Week 3 and Subsequent Weeks	Week 3 and Week 6
L*	**^α^** C	72.31	−2.1 ***	0.4 ***	−0.1***
	**^α^** D1	73.80	−6.3 ***	2.41 ***	3.95 ***
	**^α^** D2	72.55	−9.25 ***	2.79 ***	3.30 ***
a *	**^α^** C	−2.79	4.5 ***	−0.4 ***	0.5 ***
	**^α^** D1	−2.75	8.57 ***	7.64 ***	7.65 ***
	**^α^** D2	−1.68	10.19 ***	7.08 ***	6.42 ***
b *	**^α^** C	61.99	0.4 ***	0.4 ***	−1.0 ***
	**^α^** D1	58.48	3.8 ***	−0.02 ***	4.97 ***
	**^α^** D2	59.81	6.1 ***	4.98 ***	5.18 ***

**^α^** C = control diet with 0% spirulina; **^α^** D1 = control diet supplemented with 1.5% spirulina; **^α^** D2 = control diet supplemented with 2.5% spirulina; *** = *p* < 0.0001.

**Table 8:** Effect of dietary incorporation of spirulina on egg yolk cholesterol concentration.

	Treatment			SEM	*p-Value*		
	C	D1	D2		*Trt*	*W*	Trt * W
Egg yolk total cholesterol, mg/g of yolk	14.35 ± 0.88	13.89 ± 1.21	14.39 ± 1.23	0.35	NS	*	NS

C = control diet with 0% spirulina; D1 = control diet supplemented with 1.5% spirulina; D2 = control diet supplemented with 2.5% spirulina; SEM = standard error of the mean; Trt = treatment; W = week; Trt * W = treatment–week interaction; * = *p* < 0.05; NS = *p* ≥ 0.05; ^a^: Means in the same row with the same superscripts are not significantly different (*p* ≥ 0.05).
